# Knowledge, attitudes, and practices toward obesity and their determinants: a cross-sectional study

**DOI:** 10.3389/fpubh.2026.1768449

**Published:** 2026-03-23

**Authors:** Othman AlOmeir, Faisal Almutairi, Matar Mamdouh Alrouleh, Mohammed Baeissa, Syed Mohammed Basheeruddin Asdaq, Rafiulla Gilkaramenthi, Majidah Abdulrazaq AlAnazi, Fayez Mohammad Alasmari, Mohammed Sharique Ahmed Quadri, Shahabuddin T. Shaikh, Faiqa Nausheen

**Affiliations:** 1 Department of Clinical Pharmacy, College of Pharmacy, Shaqra University, Shaqra, Saudi Arabia; 2 Department of Pharmacy Practice, College of Pharmacy, AlMaarefa University, Riyadh, Saudi Arabia; 3 Research Center, Deanship of Scientific Research and Post-Graduate Studies, AlMaarefa University, Riyadh, Saudi Arabia; 4 Department of Emergency Medical Services, College of Applied Sciences, AlMaarefa University, Riyadh, Saudi Arabia; 5 Department of Nursing, King Abdulaziz Medical City, Riyadh, Saudi Arabia; 6 Department of Pharmacy, King Abdulaziz Medical City, Riyadh, Saudi Arabia; 7 Department of Basic Medical Sciences, College of Medicine, Almaarefa University, Riyadh, Saudi Arabia

**Keywords:** practices, attitudes, knowledge, obesity, public health, sociodemographic factors

## Abstract

**Background:**

Obesity remains a major global health challenge and a significant contributor to chronic diseases. This study aimed to assess knowledge, attitudes, and practices (KAP) related to obesity and to identify sociodemographic and clinical factors influencing these domains among individuals with obesity.

**Methods:**

A cross-sectional survey was conducted using a convenience, non-probability sampling method among individuals with obesity recruited from hospitals, health clinics, and community settings. Obesity-related knowledge, attitudes, and practices were assessed, and multinomial regression analyses were performed to identify factors associated with KAP outcomes.

**Results:**

A total of 419 participants were included, with a nearly equal gender distribution and a predominantly young adult population, most of whom had university-level education. Overall, 45.3% demonstrated satisfactory knowledge, 42.2% exhibited positive attitudes, and 43.0% reported effective practices regarding obesity management. Increasing age (OR = 1.361, 95% CI: 1.134–1.635) and higher education (OR = 2.417, 95% CI: 1.575–3.710) were significantly associated with satisfactory knowledge. Male participants were more likely to exhibit negative attitudes than females (OR = 1.909, 95% CI: 1.141–3.195), while individuals diagnosed with obesity were less likely to report non-satisfactory attitudes (OR = 0.495, 95% CI: 0.290–0.845). Effective practices were associated with age (OR = 1.296, 95% CI: 1.076–1.562) and obesity diagnosis (OR = 0.475, 95% CI: 0.278–0.811). Perceptions of nutritional value significantly influenced both knowledge (OR = 0.741, 95% CI: 0.571–0.961) and practices (OR = 0.384, 95% CI: 0.288–0.512).

**Conclusion:**

The findings highlight the role of key sociodemographic and clinical factors—particularly age, education, and obesity diagnosis—in shaping obesity-related knowledge, attitudes, and practices. Targeted educational and behavioral interventions, especially among younger individuals and males, along with improved awareness of nutritional value, may enhance obesity management outcomes.

## Introduction

Obesity is a complex, chronic disease influenced by multiple interacting factors, including genetic, metabolic, psychological, sociocultural, and environmental determinants (1, 2). In 2013, the American Medical Association (AMA) formally recognized obesity as a chronic disease, a position further reinforced by the World Obesity Federation in its 2017 statement, emphasizing the need for early detection and effective management strategies (2, 3). Despite growing awareness, the global prevalence of obesity continues to rise, making it a major public health concern worldwide (4). In Saudi Arabia (SA), the prevalence of obesity remains alarmingly high and is comparable to global trends. Current estimates indicate prevalence rates of approximately 36.9% among men and 38.0% among women, with some studies reporting rates of 30.0% for men and 44.4% for women (5). Evidence from studies conducted in Saudi Arabia has demonstrated that obesity is significantly associated with several chronic conditions, including hypertension, diabetes mellitus, and hypercholesterolemia (6). Obesity is linked to a wide range of adverse health outcomes. It substantially increases the risk of cardiovascular diseases (7), contributes to the development of various cancers, and is associated with disorders of the digestive system (8). In addition to its physical health consequences, obesity can also lead to significant psychological distress and negatively affect quality of life and daily functioning (9).

Social and environmental factors (10), psychological factors (11), and genetic factors (12) are risk factors for obesity. The high prevalence of obesity can be attributed to multiple factors. First, the consumption of high-fat foods has significantly increased as living standards have improved (13). Moreover, urbanization has led to a higher percentage of jobs requiring less physical strength (14). More specifically, a low resting metabolic rate is the primary cause of obesity (15). According to one study, individuals who eat irregularly have a higher risk of obesity than the general population (16). A positive psychological attitude can also help in the treatment of obesity (17). Further, weight loss is aided by both physical activity and a healthy diet.

The Ministry of Health’s (MOH) guidelines, which seek to create a comprehensive guiding development program for clinicians, including clinical pharmacists and family medicine consultants, to improve the prevention, early diagnosis, and management of obesity, have made managing obesity in SA a key component (1).

Minimizing or reducing the prevalence of obesity in the Saudi population depends on the knowledge, attitudes, and practices (KAP) of the general people. The Knowledge, Attitude, and Practice (KAP) model is a widely used framework in public health research to evaluate individuals’ awareness, perceptions, and behaviors regarding health conditions and preventive practices. The model assumes that knowledge influences attitudes, which in turn shape health-related practices and behavioral outcomes. KAP studies are particularly valuable for identifying knowledge gaps, misconceptions, and behavioral barriers that may influence disease prevention and management strategies. In the context of obesity, assessing KAP helps to understand how individuals perceive obesity, how they respond to risk factors, and whether they adopt healthy lifestyle behaviors such as balanced nutrition and regular physical activity (18, 19).

Although many studies have examined the prevalence and biomedical risk factors of obesity in Saudi Arabia, relatively fewer have explored knowledge, attitudes, and practices (KAP) toward obesity among obese individuals, leaving a gap in behavioral and public health perspectives (20, 21). Furthermore, recent methodological guidance emphasizes the importance of standardized reporting in KAP studies to improve transparency and reproducibility. The CheckCAP (Checklist for Reporting KAP Studies) provides structured recommendations for describing study design, measurement of KAP domains, and interpretation of findings in KAP-based research (22).

To strengthen the explanatory framework of this study, the Health Belief Model (HBM) was adopted as the guiding theory. The HBM suggests that individuals’ health-related behaviors are influenced by their perceived susceptibility to a condition, perceived severity of its consequences, perceived benefits of action, and perceived barriers to adopting healthy practices. By applying the HBM, this study provides a theoretical basis to better understand the relationship between knowledge, attitudes, and practices regarding obesity and to explain why certain behavioral patterns are observed in the Saudi population (23).

The current study’s goal was to assess obese people’s knowledge, attitudes, and practices about obesity. Furthermore, this study evaluated several factors, such as sociodemographic characteristics, the existence of chronic illnesses, and other factors that impact the development of obesity and their impact on knowledge, attitudes, and practices.

## Subjects and methods

### Study design

This observational cross-sectional study was conducted in Saudi Arabia between September and November 2023. Participants were recruited using a convenience, nonprobability sampling method. Although this sampling approach facilitated rapid recruitment across multiple settings, it may introduce selection bias and limit the generalizability of the findings to the broader Saudi Arabian population. However, efforts were made to recruit participants from multiple settings, including hospitals, clinics, community events, and health camps, to enhance the diversity of the sample and partially mitigate this limitation. Eligibility criteria included being aged 18 years or older, understanding the study objectives, providing informed consent, and having obesity defined as a body mass index (BMI) ≥ 30 kg/m^2^. Participants reported their height and weight, from which BMI was calculated using the standard formula (weight in kilograms divided by height in meters squared). BMI is widely used in epidemiological and population-based studies as a practical and cost-effective indicator of obesity, although it does not directly measure body fat distribution. Participants were provided with links to an online questionnaire hosted on Google Forms. The questionnaire was primarily self-administered; however, interviewer assistance was available if participants requested clarification or technical help. At the beginning of the questionnaire, the study’s objectives were explained, and informed consent was obtained, ensuring voluntary participation. For participants unable to read, the informed consent process was conducted verbally.

The study was guided by the Health Belief Model (HBM), which posits that individuals’ health behaviors are influenced by their perceptions of susceptibility, severity, benefits, and barriers. The questionnaire domains on knowledge, attitudes, and practices were mapped conceptually to these constructs, ensuring that the assessment of obesity-related behaviors was grounded in a behavioral framework.

The sample size was calculated using a 5% margin of error and a 95% confidence level, and an expected prevalence of 50% (a conservative assumption when prevalence is unknown), resulting in a required sample size of 377, as determined by the Raosoft sample size calculator.^1^ Ultimately, 419 participants were recruited and included in the final data analysis.

To enhance the transparency and reporting quality of this Knowledge–Attitude–Practice (KAP) study, the methodology and reporting were aligned with the recommendations of the CheckCAP (Checklist for Reporting KAP Studies) framework. The checklist provides structured guidance on reporting study design, sampling strategy, questionnaire development, scoring procedures, and interpretation of KAP domains. Relevant components of the checklist were considered during the preparation of the study protocol, questionnaire design, and reporting of results to ensure methodological clarity and reproducibility (22).

Because a convenience sampling strategy was used, the findings should be interpreted with caution when generalizing to the broader Saudi population. However, recruitment from multiple healthcare and community settings helped enhance sample diversity and partially improve representativeness (22).

### Ethical considerations

The study protocol was approved by the Institutional Ethical Committee of AlMaarefa University (IRB23-091). The research adhered to the STROBE (Strengthening the Reporting of Observational Studies in Epidemiology) guidelines. Informed consent was obtained from all participants, emphasizing confidentiality, voluntariness, and the right to withdraw at any stage.

### Questionnaire development

The questionnaire was developed in English by the research team following a structured review of published KAP studies related to obesity and lifestyle behaviors (18, 19, 21, 24) and guided by recommendations for the development and reporting of KAP survey instruments (22). Items were designed to measure knowledge, attitudes, and practices related to obesity risk factors, prevention strategies, and treatment options. It was later translated into Arabic using back-translation to ensure linguistic and conceptual accuracy.

In addition, the structure of the questionnaire was informed by the Health Belief Model (HBM). Items within the knowledge domain corresponded to perceived susceptibility and awareness of risk factors; attitude items reflected perceived severity, benefits, and barriers; and practice items assessed self-efficacy and cues to action in adopting healthy behaviors. This mapping strengthened the theoretical grounding of the instrument and allowed for the interpretation of KAP findings in relation to behavioral constructs (23).

Operational Definitions of KAP Domains: In accordance with the CheckCAP recommendations, the three domains of the questionnaire were operationally defined prior to data collection. “Knowledge” referred to participants’ factual understanding of obesity risk factors, complications, and management strategies. “Attitude” referred to participants’ beliefs, perceptions, and evaluations related to obesity prevention and treatment. “Practice” referred to self-reported behaviors related to diet, physical activity, medication use, and lifestyle modifications aimed at weight control. These operational definitions ensured conceptual clarity and facilitated consistent interpretation of the KAP outcomes (22, 25).

#### The instrument consisted of six domains

Sociodemographic Characteristics: Gender, age, education level, location, and employment status (self-reported, categorical items).

Clinical Status: Obesity diagnosis, family history, anxiety, sleep patterns, perceived impact of COVID-19, anti-obesity medications, weight-reduction surgery, and comorbidities. These were assessed through yes/no and multiple-choice self-reported questions. For example, participants were asked to indicate whether they had undergone any anti-obesity surgery (Yes/No), and to specify any anti-obesity medications they were using, with options such as Liraglutide, Naltrexone-Bupropion, Lorcaserin, Phentermine-Topiramate, Orlistat, Metformin, acid reflux medications, and natural products.

General Information: Body shape perception, attitudes toward weight management, engagement in leisure activities, and importance of nutritional value in food selection. These were measured using Likert-scale and multiple-choice questions. For example, participants perceived body shape was assessed with options such as: “Skinny,” “A little thin,” “Normal,” “A little fat,” and “Very fat.” Attitudes toward weight management were measured using options such as: “Indifference,” “Busy,” “Struggle,” “Goal,” and “Health.”

Knowledge Assessment: The knowledge domain assessed participants’ understanding of factors associated with obesity and its management. It included items on the role of unhealthy dietary patterns, such as consumption of foods high in fats and sugars, as contributors to obesity, as well as the impact of persistent stress as a risk factor. It also evaluated knowledge of lifestyle modifications, specifically the effectiveness of maintaining regular physical activity and a healthy diet in managing obesity. In addition, the domain explored awareness of smoking as a contributor to obesity-related complications and recognition of approved anti-obesity medications that may enhance weight loss when combined with exercise and dietary interventions.

Attitude Assessment: The attitude domain explored participants’ perceptions related to diet, physical activity, self-care, and pharmacological approaches to obesity management. It assessed beliefs about whether healthy individuals can consume both healthy and unhealthy diets, as well as perceptions regarding the need to maintain physical activity once a normal body mass index (BMI) is achieved. It also examined attitudes toward the importance of self-care, particularly whether individuals with obesity should be knowledgeable about self-management strategies to reduce the risk of complications. In addition, it addressed views on pharmacological interventions, focusing on whether anti-obesity medications are perceived as unsafe and less effective compared to lifestyle modifications such as exercise and adherence to a healthy diet. These items were assessed using a 5-point Likert scale (1 = Strongly Disagree to 5 = Strongly Agree), with all statements phrased positively to avoid confusion.

Practice Assessment: The practice domain evaluated participants’ engagement in behaviors related to obesity prevention and management. It included dietary practices such as the consumption of caffeinated drinks, frequency of fast-food intake, and adherence to a structured food regimen for weight reduction. It also assessed lifestyle practices, including participation in physical exercise for weight management and the maintenance of a healthy lifestyle. In addition, the domain examined the use of tobacco and the practice of taking anti-obesity medications as part of weight control strategies. To accurately measure these behaviors, items included both frequency-based multiple-choice questions (e.g., “How often do you consume caffeinated drinks?”) and Likert-scale questions assessing agreement with statements related to lifestyle adherence (scored 1 to 5). Total practice scores were computed by summing these values to reflect overall adherence.

### Questionnaire translation and validation process

The questionnaire was translated into Arabic using a back-translation process to ensure both linguistic and conceptual accuracy. Two independent bilingual translators performed the forward and backward translations, and discrepancies were resolved through consensus to ensure semantic equivalence between the English and Arabic versions. Validation included:

Content Validity: The instrument was reviewed by six experts from pharmacology, community medicine, and pharmacy, and subsequently revised based on their feedback. The Content Validity Index (CVI) was 0.82, reflecting an acceptable level of expert agreement. The experts also assessed whether the questionnaire items adequately represented the constructs of knowledge, attitudes, and practices related to obesity.

Criteria Validity: Aligned with established literature and validated instruments to ensure relevance. Items were cross-checked against validated KAP questionnaires from previous obesity-related studies; items not supported by prior evidence were removed. This step helped ensure that the questionnaire items were conceptually consistent with previously validated measures used in obesity-related behavioral research.

Internal Consistency: Cronbach’s alpha of 0.726 confirmed satisfactory reliability. According to widely accepted benchmarks, *α* values of 0.70 or higher are considered satisfactory for newly developed or exploratory instruments, whereas values of 0.80 or higher are desirable for more established scales (26). These findings support the reliability of the questionnaire for assessing KAP toward obesity. Internal consistency reliability indicates that the items within each domain measure related aspects of the same underlying construct.

Pilot Testing: A pilot study was conducted among 20 participants’ representative of the target population to evaluate the clarity, comprehension, and relevance of questionnaire items. Feedback obtained during the pilot phase led to minor revisions in wording and response options to improve readability and ensure that questions were interpreted consistently. Data from the pilot study were not included in the final analysis (22).

Face Validity: The expert panel also confirmed that the mapping of items to HBM constructs was conceptually sound, enhancing the validity of using the model as a guiding framework.

Because several variables, including clinical characteristics and the KAP domains, were assessed using self-reported responses, particular attention was given to ensuring the reliability and validity of these measures. Self-reported questionnaires are widely used in behavioral and public health research because they allow efficient assessment of perceptions, attitudes, and lifestyle behaviors across large populations. However, they may be subject to recall bias or social desirability bias. The combination of expert validation, pilot testing, and internal consistency analysis was therefore employed to strengthen the credibility and reliability of the collected data.

This process ensured the questionnaire was reliable and suitable for assessing obesity-related knowledge, attitudes, and practices.

### Statistical methods

Data were analyzed using SPSS version 25 (IBM Corp., Armonk, NY, USA). Prior to analysis, the dataset was screened for completeness and consistency. Responses containing substantial missing information were excluded from the final dataset. Because the online questionnaire required completion of key items before submission, the proportion of missing data was minimal and unlikely to affect the validity of the analysis. Therefore, a complete-case analysis approach was applied for all statistical procedures.

Descriptive statistics were used to summarize participants’ sociodemographic characteristics and their levels of knowledge, attitudes, and practices (KAP) related to obesity. Continuous variables were presented as means and standard deviations, while categorical variables were summarized using frequencies and percentages. Pearson correlation analysis was conducted to examine the relationships among the knowledge, attitude, and practice domains. Each item within these domains was assessed using a five-point Likert scale (strongly agree = 5, agree = 4, neutral = 3, disagree = 2, strongly disagree = 1). For each participant, the mean score of all items within each domain was calculated. Subsequently, the overall mean score across all participants was computed for each domain, yielding mean scores of 4.06/5 for knowledge, 3.27/5 for attitude, and 3.16/5 for practice. Based on these aggregate mean scores, participants were categorized as having satisfactory knowledge, positive attitudes, or effective practices if their individual scores exceeded the respective domain means. This classification approach is commonly employed in KAP studies to facilitate interpretation of behavioral outcomes and enable comparison with previous research (27).

Multinomial regression analysis was performed to identify factors associated with knowledge, attitude, and practice levels while adjusting for relevant sociodemographic variables. In addition, Chi-square tests were used to examine associations between sociodemographic characteristics and categorical outcomes, including adequate versus inadequate knowledge, positive versus negative attitudes, effective versus ineffective practices, and levels of perceived barriers. A *p*-value < 0.05 was considered statistically significant.

## Results

### Sociodemographic, clinical, and general characteristics of the participants

Table 1 presents the sociodemographic profile of the study participants, covering gender, age, education, location, and employment status (*n* = 419). The sample is almost evenly distributed between males (49.9%) and females (50.1%). The largest age group is 25–30 years (30.5%), though participants from other age ranges are also well represented. Most participants have a university-level education (67.8%) and reside in the Middle region (74.5%). Regarding employment, the majority work in the governmental or private sector (43.4%), followed by the unemployed group (27.7%).

**Table 1:** Characteristics of the participants.

Characteristics	Variables	Frequency (percentage) *n* (%)
Gender	Male	209 (49.9)
	Female	210 (50.1)
Age (years)	18–24	92 (22.0)
	25–30	128 (30.5)
	31–35	65 (15.5)
	36–40	73 (17.4)
	>40	61 (14.6)
Education	Intermediate school certificate only	12 (2.9)
	High school certificate only	109 (26.0)
	University bachelor or diploma certificates	284 (67.8)
	Higher studies certificates such as master’s and PhD	14 (3.3)
Location	Middle region	312 (74.5)
	Western region	38 (9.1)
	Eastern region	34 (8.1)
	Southern region	14 (3.3)
	Northern region	21 (5.0)
Employment status	Unemployed	116 (27.7)
	Student	92 (22.0)
	Free business	26 (6.2)
	Governmental or private sector	182 (43.4)
	Retired	3 (0.7)

Although all participants met the BMI criterion for obesity, only 29.1% reported having received a formal medical diagnosis of obesity, while 70.9% had not been diagnosed by a healthcare professional, and only half had a family history of it (50.1%) (Supplementary Table 1). Stress or anxiety and sleep issues were reported by 28.4 and 34.1%, respectively. The majority (68.7%) reported a significant COVID-19 impact. Anti-obesity surgery was rare (3.8%), and medications like Metformin (14.31%) were used by a small proportion. Common co-morbidities included diabetes (28.2%), cardiac diseases (26.3%), and hypertension (22%).

Most participants perceive themselves as “a little fat” (50.8%) or “very fat” (30.5%). Regarding weight management, the largest group struggles with it (37.7%). In terms of entertainment, social service activities were most common (41.5%), while participation in sports was minimal (2.9%). When selecting food based on nutritional value, most participants “rarely” (38.7%) or “often” (34.6%) consider it (Supplementary Table 2).

### Status of knowledge, attitude, and practice toward obesity

While 45.3% of participants demonstrated satisfactory knowledge, the majority (54.7%) had non-satisfactory knowledge (Table 2). Regarding attitude, 42.2% had a positive attitude toward obesity, while 57.8% held a negative attitude. In terms of practices, 43.0% of participants engaged in effective practices, whereas 57.0% showed ineffective practices related to obesity management.

**Table 2:** Status of knowledge, attitude, and practices on obesity.

Outcome	Variables	Frequency (percentage) *n* (%)
Knowledge	Satisfactory	190 (45.3)
	Non-satisfactory	229 (54.7)
Attitude	Positive	177 (42.2)
	Negative	242 (57.8)
Practice	Effective	180 (43.0)
	Ineffective	239 (57.0)

### Association between KAP domains and sociodemographic characteristics

Age and education are significantly associated with knowledge status, while gender and location are not. Younger participants (18–24) and those with lower education levels have poorer knowledge. Higher education is significantly associated with satisfactory knowledge (*p* < 0.001). However, the association between employment status and knowledge was not significant (*p* = 0.050) (Table 3).

**Table 3:** Comparison of KAP domains with sociodemographic characteristics.

Characteristics	Knowledge status, *n* (%)			Attitude status, *n* (%)			Practice status, *n* (%)		
	Satisfactory	Non-satisfactory	*p* value	Positive	Negative	*p* value	Effective	Ineffective	*p* value
**Gender**									
Male	103 (49.3)	106 (50.7)	0.106	71 (34.0)	138 (66.0)	0.001	77 (36.8)	132 (63.2)	0.012
Female	87 (41.4)	123 (58.6)		106 (50.5)	104 (49.5)		103 (49.0)	107 (51.0)	
**Age (years)**									
18–24	28 (30.4)	64 (69.6)	0.001	39 (42.4)	53 (57.6)	0.310	32 (34.8)	60 (65.2)	0.004
25–30	64 (50.0)	64 (50.0)		60 (46.9)	68 (53.1)		45 (35.2)	83 (64.8)	
31–35	21 (32.3)	44 (67.7)		31 (47.7)	34 (52.3)		29 (44.6)	36 (55.4)	
36–40	41 (56.2)	32 (43.8)		25 (34.2)	48 (65.8)		37 (50.7)	36 (49.3)	
>40	36 (59.0)	25 (41.0)		22 (36.1)	39 (63.9)		37 (60.7)	24 (39.3)	
**Education**									
Intermediate school	1 (8.3)	11 (91.7)	0.000	8 (66.7)	4 (33.3)	0.018	4 (33.3)	8 (66.7)	0.918
High school	33 (30.3)	76 (69.7)		55 (50.5)	54 (49.5)		48 (44.0)	61 (56.0)	
Bachelor or diploma	149 (52.5)	135 (47.5)		106 (37.3)	178 (62.7)		122 (43.0)	162 (57.0)	
Master and PhD	7 (50.0)	7 (50.0)		8 (57.1)	6 (42.9)		6 (42.9)	8 (57.1)	
**Location**									
Middle region	144 (46.2)	168 (53.8)	0.389	113 (36.2)	199 (63.8)	0.001	133 (42.6)	179 (57.4)	0.340
Western region	12 (31.6)	26 (68.4)		19 (50.0)	19 (50.0)		12 (31.6)	26 (68.4)	
Eastern region	17 (50.0)	17 (50.0)		24 (70.6)	10 (29.4)		19 (55.9)	15 (44.1)	
Southern region	8 (57.1)	6 (42.9)		9 (64.3)	5 (35.7)		6 (42.9)	8 (57.1)	
Northern region	9 (42.9)	12 (57.1)		12 (57.1)	9 (42.9)		10 (47.6)	11 (52.4)	
**Employment status**									
Unemployed	49 (42.2)	67 (57.8)	0.050	57 (49.1)	59 (50.9)	0.311	53 (45.7)	63 (54.3)	0.201
Student	35 (38.0)	57 (62.0)		40 (43.5)	52 (56.5)		32 (34.8)	60 (65.2)	
Free business	8 (30.8)	18 (69.2)		12 (46.2)	14 (53.8)		13 (50.0)	13 (50.0)	
Governmental or private sector	97 (53.3)	85 (46.7)		67 (36.6)	115 (63.2)		82 (45.1)	100 (54.9)	
Retired	1 (33.3)	2 (66.7)		1 (33.3)	2 (66.6)		0 (0.0)	3 (100.0)	
**Diagnosis by the health practitioner**									
Yes	64 (52.5)	58 (47.5)	0.061	61 (50.0)	61 (50.0)	0.039	66 (54.1)	56 (45.9)	0.003
No	126 (42.4)	171 (57.6)		116 (39.1)	181 (60.9)		114 (38.4)	183 (61.6)	
**Family history of obesity**									
Yes	101 (48.1)	109 (51.9)	0.257	90 (42.9)	120 (57.1)	0.799	85 (40.5)	125 (59.5)	0.303
No	89 (42.6)	120 (57.4)		87 (41.6)	122 (58.4)		95 (45.5)	114 (54.5)	
**Stress or anxiety**									
Yes	63 (52.9)	56 (47.1)	0.049	43 (36.1)	76 (63.9)	0.111	56 (47.1)	63 (52.9)	0.286
No	127 (42.3)	173 (57.7)		134 (44.7)	166 (55.3)		124 (41.3)	176 (58.7)	
**Sleep issues**									
Yes	65 (45.5)	78 (54.5)	0.974	66 (46.2)	77 (53.8)	0.243	58 (40.6)	85 (59.4)	0.475
No	125 (45.3)	151 (54.7)		111 (40.2)	165 (59.8)		122 (44.2)	154 (55.8)	
**COVID-19 impact**									
Yes	145 (50.3)	143 (49.7)	0.002	108 (37.5)	180 (62.5)	0.004	120 (41.7)	168 (58.3)	0.428
No	45 (34.4)	86 (65.6)		69 (52.7)	62 (47.3)		60 (45.8)	71 (54.2)	
**Surgery**									
Yes	6 (37.5)	10 (62.5)	0.520	9 (56.3)	7 (43.8)	0.247	10 (62.5)	6 (37.5)	0.107
No	184 (45.7)	219 (54.3)		168 (41.7)	235 (58.3)		170 (42.2)	233 (57.8)	

Gender, age, education, and location significantly impact attitude status. Males show more non-satisfactory attitudes than females (*p* = 0.001). Younger participants (18–24) and those with lower education levels exhibit more non-satisfactory attitudes. The participants from the middle region have a higher proportion of non-satisfactory attitudes (*p* = 0.001). Employment status has little effect (Table 3).

Gender and age affect practice status. Males have a higher proportion of non-satisfactory practice status compared to females (*p* = 0.012). Younger participants (18–24) exhibit more non-satisfactory practices. Education and location do not significantly impact practice status, though students show poorer practice outcomes compared to those in government or private sector jobs (Table 3).

Participants diagnosed with obesity by a health practitioner show slightly better knowledge, though the association is nearly significant. No significant associations are found with family history, sleep issues, or surgery history. Stress or anxiety significantly improves knowledge status (*p* = 0.049), and participants impacted by COVID-19 show better knowledge (*p* = 0.002).

For attitude status, participants diagnosed with obesity have more satisfactory attitudes (*p* = 0.039), while no significant associations are found with family history, sleep issues, or surgery history. Stress or anxiety does not affect attitude status, and those impacted by COVID-19 exhibit poorer attitudes (*p* = 0.004).

Regarding practice status, obesity diagnosis is significantly associated with better practices (*p* = 0.003). Stress, sleep issues, family history, and surgery history do not show significant associations.

### Correlation between knowledge, attitude, and practices

Table 4 presents significant correlations between knowledge, attitude, and practice status. Knowledge and attitude status have a negative correlation (*r* = −0.148, *p* = 0.002), meaning that as knowledge increases, attitudes tend to become less favorable, though weakly. Knowledge and practice status are positively correlated (*r* = 0.246, *p* = 0.000), indicating that higher knowledge leads to more satisfactory practices. The negative correlation between attitude and practice status (*r* = −0.118, *p* = 0.016) shows that more positive attitudes are associated with less satisfactory practices. The strongest correlation is between knowledge and practice status.

**Table 4:** Correlation of knowledge, attitude, and practices of participants toward obesity.

Outcome	Variables	Knowledge	Attitude	Practice
Knowledge	Pearson correlation	1	−0.148^a^	0.246^a^
	Sig. (2-tailed)	–	0.002	0.000
Attitude	Pearson correlation	–	1	−0.118^b^
	Sig. (2-tailed)	–	–	0.016
Practice	Pearson correlation	–	–	1
	Sig. (2-tailed)	–	–	–

a Correlation is significant at the 0.01 level (2-tailed).

b Correlation is significant at the 0.05 level (2-tailed).

### Factors influencing the KAP domains of the participants

Table 5 presents the multinomial regression analysis for factors influencing satisfactory knowledge, positive attitude, and effective practice statuses. Age significantly influences knowledge, with older participants more likely to have satisfactory knowledge (*p* = 0.001, OR = 1.361, 95% CI: 1.134–1.635). Education level also plays a key role (*p* = 0.000, OR = 2.417, 95% CI: 1.575–3.710), with higher education linked to satisfactory knowledge. Perception of nutritional value (*p* = 0.024, OR = 0.741, 95% CI: 0.571–0.961) influences knowledge, with higher perceived nutritional knowledge associated with satisfactory knowledge. Gender, family history, and sleep issues do not significantly impact knowledge status.

**Table 5:** Multinomial regression analysis for determining factors that influence the outcome variables.

Factors	Knowledge ^a^				Attitude ^b^				Practice ^c^			
	Sig.	Odds ratio	95% confidence interval for OR		Sig.	Odds ratio	95% confidence interval for OR		Sig.	Odds ratio	95% confidence interval for OR	
			Lower bound	Upper bound			Lower bound	Upper bound			Lower bound	Upper bound
Gender	0.210	0.726	0.440	1.198	0.014	1.909	1.141	3.195	0.270	1.338	0.797	2.246
Age	0.001	1.361	1.134	1.635	0.811	0.978	0.817	1.171	0.006	1.296	1.076	1.562
Education	0.000	2.417	1.575	3.710	0.096	0.701	0.462	1.064	0.428	1.191	0.773	1.833
Location	0.101	1.191	0.967	1.467	0.003	1.372	1.111	1.695	0.385	1.103	0.884	1.377
Employment	0.088	0.837	0.682	1.027	0.974	1.004	0.814	1.237	0.230	0.879	0.712	1.085
Obesity diagnosed	0.533	0.849	0.508	1.419	0.010	0.495	0.290	0.845	0.006	0.475	0.278	0.811
Family history of obesity	0.182	0.730	0.459	1.159	0.430	1.209	0.754	1.938	0.157	1.417	0.875	2.296
Stress or anxiety	0.104	0.647	0.383	1.093	0.042	1.774	1.020	3.086	0.304	0.750	0.434	1.298
Sleep issues	0.672	1.117	0.669	1.867	0.253	0.738	0.438	1.243	0.498	1.208	0.699	2.086
COVID-19 impact	0.011	0.530	0.325	0.863	0.158	1.416	0.874	2.294	0.860	0.956	0.576	1.584
Surgery	0.986	1.010	0.311	3.280	0.448	1.615	0.469	5.570	0.184	0.438	0.130	1.481
Perceived body shape	0.193	0.813	0.596	1.110	0.567	1.095	0.802	1.496	0.273	0.832	0.598	1.157
Perceived attitude	0.033	1.192	1.015	1.400	0.004	0.787	0.669	0.926	0.551	1.053	0.888	1.249
Entertainment	0.037	1.175	1.010	1.367	0.004	0.794	0.679	0.927	0.976	1.002	0.858	1.171
Nutritional value	0.024	0.741	0.571	0.961	0.000	1.646	1.260	2.150	0.000	0.384	0.288	0.512

a Non-satisfactory knowledge.

b Negative attitude.

c Ineffective practice.

^a,b,c^ reference categories for knowledge, attitude, and practice.

For attitude status, gender (*p* = 0.014, OR = 1.909, 95% CI: 1.141–3.195) significantly influences attitudes, with females more likely to have positive attitudes. Perception of attitude (*p* = 0.033, OR = 1.192, 95% CI: 1.015–1.400) and obesity diagnosis (*p* = 0.010, OR = 0.495, 95% CI: 0.290–0.845) also significantly affect attitudes. Entertainment (*p* = 0.037, OR = 1.175, 95% CI: 1.010–1.367) and nutritional value perception (*p* = 0.000, OR = 1.646, 95% CI: 1.260–2.150) significantly influence attitudes.

For practice status, age (*p* = 0.006, OR = 1.296, 95% CI: 1.076–1.562) and obesity diagnosis (*p* = 0.006, OR = 0.475, 95% CI: 0.278–0.811) are significant factors. A positive perception of nutritional value (*p* = 0.000, OR = 0.384, 95% CI: 0.288–0.512) is also linked to effective practices. Gender, family history, and sleep issues do not significantly affect practice status.

## Discussion

This study assessed individuals’ knowledge, attitudes, and practices (KAP) toward obesity and the factors influencing these variables. The sociodemographic and clinical characteristics of the participants provide insights into their engagement with obesity-related issues, and the analysis highlights areas for intervention.

The sociodemographic profile showed a balanced gender distribution, with many participants being university-educated and from the middle region of Saudi Arabia. The 25–30 age group was the largest, reflecting a young, urbanized population. Education emerged as a strong determinant of satisfactory knowledge, with individuals having higher education levels demonstrating better knowledge, consistent with global studies (28).

Regarding attitudes, males had more non-satisfactory attitudes than females, reflecting cultural influences where men may place less emphasis on weight management. Regional variations showed poorer attitudes toward obesity in the Middle Region, possibly due to local health policies or socio-cultural factors (29).

Clinical status, including obesity diagnosis, family history, and mental health factors like stress and anxiety, significantly influenced participants’ KAP. Although obesity diagnosis was not linked to knowledge, it was associated with more favorable attitudes and practices, reflecting psychological motivation to adopt healthier behaviors (30). Stress or anxiety positively influences knowledge, suggesting that emotional distress may encourage individuals to seek information about their condition, which aligns with other findings (31). However, stress did not affect attitude or practice status, which warrants further investigation into the relationship between psychological factors and health behavior.

The study found that participants’ KAP toward obesity was not aligned. While a moderate positive correlation existed between knowledge and practices, the negative correlation between knowledge and attitudes suggests that individuals may be aware of obesity risks but struggle to internalize the emotional and social aspects of obesity management (32). The inverse relationship between attitude and practice may reflect elements of cognitive dissonance, where individuals hold favorable attitudes toward obesity prevention in principle but fail to align these with actual behaviors due to competing priorities, convenience, or cultural habits (33). Alternatively, social desirability bias may have influenced reported attitudes, as participants may have expressed socially acceptable views about diet and activity without engaging in corresponding practices. Recognizing these psychosocial mechanisms is critical, as future interventions must not only shape attitudes but also provide structural and motivational supports that facilitate the translation of intentions into action (34). Younger participants had less favorable attitudes, aligning with findings from other studies showing more negative perceptions of obesity among younger populations (35). Age significantly improved practice status, with older participants more likely to engage in satisfactory practices, in line with global patterns where older individuals tend to adopt more health-conscious behaviors due to increased awareness of age-related health risks (36).

The multinomial regression analysis identified key factors influencing non-satisfactory knowledge, attitude, and practice statuses. Age was a significant factor influencing both knowledge and practice statuses, with older participants more likely to demonstrate satisfactory outcomes. This finding is consistent with other studies suggesting that age-related improvements in health behaviors come from greater life experience and accumulated knowledge (34). Education also played a crucial role, particularly in improving knowledge, with those holding higher educational qualifications more likely to have satisfactory knowledge, as supported by global studies (36).

The multinomial regression findings provide actionable insights for public health. For example, older age groups showing higher odds of satisfactory practices suggest that interventions should particularly target younger populations, who remain less engaged in obesity prevention. Similarly, higher educational attainment as a predictor of knowledge underscores the importance of integrating obesity-related health education into primary and secondary school curricula to bridge gaps among less-educated groups. Gender disparities in attitudes further indicate that public health policy should tailor obesity campaigns to engage men more effectively, potentially through workplace wellness programs or male-focused health initiatives. Thus, odds ratios not only quantify statistical associations but also highlight priority groups for targeted obesity-prevention strategies (37).

Gender and perception of one’s attitude significantly influenced attitude status, with males exhibiting poorer attitudes toward obesity, consistent with studies from the U.S. and Europe (28). The influence of entertainment on attitude was an interesting finding; increased engagement in entertainment activities might encourage a more passive and less health-conscious lifestyle, negatively impacting attitudes toward obesity management (38). The gender differences observed—where men demonstrated poorer attitudes toward obesity management—can be understood through a gendered lens. In Saudi Arabia, cultural and social norms may place less emphasis on men’s body weight, while women often face stronger social pressures regarding appearance and health behaviors (39). Additionally, women may be more engaged with healthcare systems due to reproductive health visits, providing more opportunities for health education. Men, conversely, may have fewer touchpoints with preventive healthcare and may prioritize occupational responsibilities over health-seeking behaviors (40). These gendered dynamics underscore the need for male-focused outreach that addresses behavioral norms and health access disparities.

For practice status, obesity diagnosis significantly improved participants’ practices, suggesting that those diagnosed with obesity are more likely to engage in proactive health behaviors, a finding supported by studies in Europe and the Middle East (41, 42). Perception of nutritional value also significantly affected practice status, underscoring the importance of nutrition literacy in improving obesity management practices (43).

The integration of the Health Belief Model (HBM) helps explain these findings more comprehensively. Participants with satisfactory knowledge reflect higher perceived susceptibility and severity regarding obesity, motivating awareness of risks. However, the weaker link between knowledge and attitudes may indicate that while individuals recognize obesity’s dangers, they perceive barriers—such as cultural norms, time constraints, or cost of healthy food—that reduce motivation to act. The stronger relationship between diagnosis and favorable practices aligns with the HBM’s principle of cues to action: once individuals are formally diagnosed, this acts as a trigger to adopt healthier behaviors. Similarly, education appears to enhance self-efficacy, supporting better knowledge and more consistent health practices. Thus, the HBM framework not only contextualizes these associations but also highlights the need for interventions targeting perceived barriers and strengthening self-efficacy in obesity management (23, 44).

Saudi Cultural and Religious Context: Cultural norms and religious practices in Saudi Arabia play a significant role in shaping obesity-related knowledge, attitudes, and practices. Traditional dietary habits, including the frequent consumption of rice, dates, and meat-rich dishes during social gatherings, contribute to high caloric intake (39). Social events often revolve around shared meals, reinforcing cultural expectations to eat abundantly. Religious teachings in Islam emphasize moderation (“do not commit excess”), which could serve as a foundation for promoting healthy eating behaviors. However, sedentary lifestyles, increased reliance on cars, and limited female access to certain physical activity venues historically contributed to low physical activity levels (40). Recent reforms expanding women’s participation in sports and public health initiatives provide opportunities to reframe cultural and religious values as facilitators of obesity prevention. Tailoring interventions to align with cultural practices—such as family-based health education and mosque-based community awareness programs—could increase acceptability and effectiveness.

Limitations: Despite its comprehensive scope, this study has several limitations. First, the cross-sectional design precludes establishing causal relationships between sociodemographic or clinical factors and obesity-related knowledge, attitudes, and practices. Longitudinal studies are therefore warranted to better understand the temporal relationships and long-term behavioral changes associated with obesity management. Second, several variables, including clinical characteristics and the KAP domains, were assessed using self-reported responses. Although self-reported questionnaires are widely used in public health research because of their feasibility and ability to capture perceptions and behaviors, they may be subject to recall bias and social desirability bias. Participants may over-report desirable behaviors, such as adherence to healthy lifestyle practices, or underreport behaviors perceived negatively. To mitigate these concerns, the questionnaire underwent expert validation, pilot testing, and internal consistency reliability assessment. Third, the use of a convenience, non-probability sampling method may introduce selection bias and limit the representativeness of the sample. Although participants were recruited from multiple healthcare and community settings to enhance diversity, the findings should be interpreted cautiously when generalizing them to the broader Saudi population or to other sociocultural contexts. Another limitation relates to the use of body mass index (BMI) to define obesity. While BMI is widely used in epidemiological studies due to its simplicity and cost-effectiveness, it does not directly measure body fat distribution or distinguish between fat mass and lean body mass. Measures of central adiposity, such as waist circumference, may provide a more accurate assessment of obesity-related health risks because they capture abdominal fat accumulation, which is strongly associated with metabolic and cardiovascular outcomes. Previous studies have shown that waist circumference may predict mortality and obesity-related health outcomes as effectively as, or in some cases better than, BMI (45, 46). Future research may therefore benefit from incorporating additional anthropometric indicators, such as waist circumference or waist-to-hip ratio, to provide a more comprehensive assessment of obesity. Finally, although the online questionnaire was designed to minimize incomplete responses by requiring answers to key questions, the possibility of missing data cannot be entirely excluded. However, the proportion of missing responses was minimal and therefore unlikely to have materially influenced the overall findings.

Implications: The findings of this study have significant implications for healthcare providers and policymakers. Understanding the factors influencing obesity-related behaviors can inform the development of more effective public health campaigns and clinical interventions. Educational programs focused on nutritional value and weight management should be integrated into routine healthcare visits, especially for those diagnosed with obesity. Tailoring strategies to sociodemographic factors such as age, education, and gender can improve the overall effectiveness of obesity management efforts.

Importantly, interventions designed within the HBM framework could address specific behavioral determinants. For example, campaigns can emphasize personal susceptibility and severity to motivate awareness, while simultaneously reducing perceived barriers (e.g., cost of healthy foods, limited access to exercise facilities). Enhancing perceived benefits and building self-efficacy through skill-based education and support groups may further strengthen sustainable obesity management behaviors.

## Conclusion

This study provides important insights into the factors influencing knowledge, attitudes, and practices toward obesity among adults. The findings highlight that demographic factors, particularly age, education level, and obesity diagnosis, play a significant role in shaping obesity-related practices. The results also demonstrate that higher knowledge does not necessarily translate into positive attitudes or appropriate practices, emphasizing the complex relationship between knowledge, attitude, and behavior in obesity management.

These findings suggest that improving obesity-related outcomes requires more than increasing awareness alone. Interventions should be designed to address behavioral and demographic differences within the population. Tailored educational and behavioral strategies that consider age and gender differences may help improve attitudes and encourage healthier practices. Strengthening health education, promoting lifestyle modification, and improving access to obesity-related counseling may contribute to more effective obesity prevention and management strategies and ultimately help reduce the burden of obesity and its associated health complications.

## Data Availability

The original contributions presented in the study are included in the article/Supplementary material, further inquiries can be directed to the corresponding author.

## Footnotes

1
http://www.raosoft.com/samplesize.html
