# Supplementary material for: Knowledge, attitudes, and practices toward obesity and their determinants: a cross-sectional study
Source: Front Public Health. 2026 Mar 23;14:1768449. doi: 10.3389/fpubh.2026.1768449 (PMC13050718; doi:10.3389/fpubh.2026.1768449)
Supplement: Supplementary file 1 [file Data_Sheet_1.pdf]

Knowledge, Attitudes, and Practices Toward Obesity and Their Determinants: A Cross-Sectional Study

Othman AlOmeir[1], Faisal Almutairi[2], Matar Mamdouh Alrouleh[2], Mohammed Baeissa[2], Syed Mohammed Basheeruddin Asdaq[2,3], Rafiulla Gilkaramenthi[4], Majidah Abdulrazaq AlAnazi[5], Fayez Mohammad Alasmari[6], Mohammed Sharique Ahmed Quadri[7,3], Shahabuddin T Shaikh[7], Faiqa Nausheen[7,*]

[1]Department of Clinical Pharmacy, College of Pharmacy, Shaqra University, Shaqra 11961, Saudi Arabia, O.k.alomeir@gmail.com
[2]Department of Pharmacy Practice, College of Pharmacy, AlMaarefa University, Dariyah, 13713, Riyadh, Saudi Arabia, sasdaq@gmail.com/sasdag@um.edu.sa (SMBA); 201120228@student.um.edu.sa (FA), 171120140@student.um.edu.sa (MMA), 172120389@student.um.edu.sa (MB)
[3]Research Center, Deanship of Scientific Research and Post-Graduate Studies, AlMaarefa University, Dariyah, 13713, Riyadh, Saudi Arabia
[4]Department of Emergency Medical Services, College of Applied Sciences, AlMaarefa University, Diriyah, 13713, Riyadh, Saudi Arabia, grafi@um.edu.sa
[5]Department of Nursing, King Abdulaziz Medical City, Riyadh, Saudi Arabia, dooda468@gmail.com (MAA)
[6]Department of Pharmacy, King Abdulaziz Medical City, Riyadh, Saudi Arabia, alasmarifa3@ngha.med.sa
[7]Department Of Basic Medical Sciences, College of Medicine, Almaarefa University, Dariyah, 13713, Saudi Arabia, mquadri@um.edu.sa (MSAQ), sshaikh@um.edu.sa (STS), nausheenfaiqa42@gmail.com (FN)

*** Correspondence:**
Faiqa Nausheen
nausheenfaiqa42@gmail.com

Supplementary Table 1: Clinical status of the participants

Characteristics	Variables	Frequency	Percentage
Obesity Diagnosed	Yes	122	29.1
	No	297	70.9
Family History of Obesity	Yes	210	50.1
	No	209	49.9
Stress or anxiety	Yes	119	28.4
	No	300	71.6
Sleep issue	Yes	143	34.1
	No	276	65.9
COVID-19 impact	Yes	288	68.7
	No	131	31.3
Any anti-obesity surgery	Yes	16	3.8
	No	403	96.2
Anti-obesity medication use	Liraglutide	3	0.71
	Naltrexone-Bupropion	3	0.71
	Lorcaserin	3	0.71
	Phentermine-Topiramate	4	0.95
	Orlistat	6	1.43
	Acid reflux medications	10	2.38
	Natural products	22	5.25
	Metformin	60	14.31
	No	308	73.50
Co-morbidity status (some patients have more than one morbidity)	Kidney diseases	6	1.4
	Other diseases	16	3.8
	Thyroid disease	19	4.5
	Arthritis	43	10.3
	Sleeping difficulties	56	13.4
	Shortness of breath	59	14.1
	Hypertension	92	22
	Cardiac diseases	110	26.3
	Diabetes	118	28.2
	No	199	47.5

Supplementary Table 2: General Information

Characteristics	Variables	Frequency	Percentage
Perceived Body shape	Skinny	8	1.9
	A little thin	4	1.0
	Normal	66	15.8
	A little fat	213	50.8
	Very fat	128	30.5
Attitude toward weight management	Indifference	46	11.0
	Busy	31	7.4
	Struggle	158	37.7
	Goal	79	18.9
	Health	105	25.1
Entertainment activities	Sleep	34	8.1
	Reading and Learning	73	17.4
	Surfing internet and playing games	87	20.8
	Shopping	39	9.3
	Social service	174	41.5
	Participation in sports	12	2.9
Selection of food based on nutritional value	Always	23	5.5
	Often	145	34.6
	Rarely	162	38.7
	Not at all	89	21.2
